# Gravitrap deployment for adult *Aedes aegypti* surveillance and its impact on dengue cases

**DOI:** 10.1371/journal.pntd.0008528

**Published:** 2020-08-07

**Authors:** Janet Ong, Chee-Seng Chong, Grace Yap, Caleb Lee, Muhammad Aliff Abdul Razak, Suzanna Chiang, Lee-Ching Ng

**Affiliations:** 1 Environmental Health Institute, National Environment Agency, Singapore; 2 Environmental Public Health Operations, National Environment Agency, Singapore; 3 School of Biological Sciences, Nanyang Technological University, Singapore; USDA-ARS Center for Medical Agricultural and Veterinary Entomology, UNITED STATES

## Abstract

House Index, Container Index, and Breteau Index are the most commonly used indices for dengue vector surveillance. However, these larval indices are a poor proxy for measuring the adult population—which is responsible for disease transmission. Information on the adult distribution and density are important for assessing transmission risk as well as for developing effective control strategies. This study introduces a new entomological index, Gravitrap *aegypti* index (GAI), which estimates the adult female *Aedes aegypti* population in the community and presents its association with dengue cases. Gravitraps were deployed across 34 treatment sites in Singapore from September 2013 to September 2016. The GAI, derived from the Gravitrap surveillance data, was analysed to investigate the spatio-temporal patterns of the *Ae*. *aegypti* population in Singapore. The index was further categorised into low, moderate, and high-risk groups and its association with dengue cases were examined. A Before-After Control Impact analysis was performed to evaluate the epidemiology impact of Gravitrap system on dengue transmission. The *Ae*. *aegypti* population exhibits a seasonal pattern, and spatial heterogeneity in *Ae*. *aegypti* abundance was observed among treatment sites. The *Ae*. *aegypti* population was also found to be unevenly distributed among floors of an apartment block, with low floors (floors 1–4) having a higher abundance of mosquitoes trapped than mid (floors 5–8) and high (floors ≥9) floors. Areas with high GAI were shown to have higher dengue case count. Gravitrap has also demonstrated to be a good dengue control tool. The contribution of cases by treatment sites to the national numbers was lower after Gravitraps deployment. The GAI, which is of better relevance to dengue transmission risk, could be recommended as an indicator for decision making in vector control efforts, and to monitor the spatio-temporal variability of the adult *Aedes* population in the country. In addition, findings from this study indicate that Gravitraps can be used as a dengue control tool to reduce dengue transmission.

## Author summary

In the absence of an effective vaccine: vector surveillance and control remain the key strategy for dengue prevention and control. The collection of the adult female *Aedes* mosquito is thus important to understand disease transmission dynamics. Information on its distribution and density are also essential for assessing transmission risk as well as for devising an effective control strategy. Here, we described a new approach to dengue vector surveillance based on adult female *Aedes* trapping using Gravitraps. Using the Gravitrap surveillance data, we derived a new entomological index, Gravitrap *aegypti* index (GAI), which estimates the adult female *Ae*. *aegypti* population in the community and presents its association with dengue cases. When analysed, the index can provide useful information on the spatio-temporal distribution of the *Ae*. *aegypti* population in the country and hence, assist planning of vector control. The GAI, which is of better relevance to dengue transmission risk, could be used as an indicator for decision making in vector control efforts, and to monitor the spatio-temporal variability of the adult *Aedes* population. In addition, findings from this study indicate that Gravitraps can be used as a dengue control tool to reduce dengue transmission.

## Introduction

Dengue fever has become the most important mosquito-borne viral disease due to its increasing frequency and magnitude of dengue epidemics as well as its high morbidity and mortality [1,2]. It is estimated that 390 million dengue infections occur each year (95% credible interval 284–528 million), with more than 125 countries known to be dengue endemic [2,3]. Dengue poses a significant public health threat globally, especially throughout the tropical and subtropical regions [4].

Dengue fever is caused by infection with one of the four dengue virus serotypes and is transmitted to humans primarily by the mosquito *Aedes aegypti* and by *Ae*. *albopictus* as a secondary vector [5,6]. Both *Ae*. *aegypti* and *Ae*. *albopictus* are known to be widely distributed in the tropical and subtropical areas of the world [7]. However, these *Aedes* species have expanded all over the world in the past decades and expanded the geographical risk of dengue outbreaks [8,9]. The dengue outbreak in Nepal in 2006 and more recently on Madeira island in 2012, are examples of epidemic driven by *Ae*. *aegypti* invasion [10,11]. The occurrence of *Ae*. *aegypti* was not reported in Nepal until 2006 when entomological survey conducted after its first dengue outbreak revealed the presence of *Ae*. *aegypti* in five major urban areas [12,13]. The outbreak on Madeira island was the first dengue epidemic in Europe since the 1920s, and it occurred in a territory where *Ae*. *aegypti* was introduced less than a decade before the outbreak [14].

Located in the tropical region of Asia-Pacific, Singapore is endemic for dengue with all four dengue serotypes co-circulating in the country at all times [15]. There is a cyclical pattern of outbreaks that oscillates between DENV-1 and DENV-2 as the predominant serotype [16]. In 2013 and 2014, Singapore experienced the largest outbreaks known to date, reporting 22,170 and 18,318 laboratory confirmed dengue cases respectively [17]. These outbreaks were associated with a switch in the predominant serotype from DENV-2 to DENV-1. The population’s susceptibility to outbreaks may have been influenced by multiple factors; an increase in human population density, improved transportation that facilitated virus dissemination, and low herd immunity resulting from sustained period of low transmission; all of which facilitated dengue transmission [18–20].

In the absence of an effective vaccine: vector surveillance and control remain the key strategy for dengue prevention and control in Singapore. Although several vector surveillance and control techniques have been developed, their utilization is limited to the sampling of the immature stages of *Aedes*, such as the eggs, larvae, and pupae [21]. Entomological indices generated from these methods–Breteau Index, Container Index, and House Index, only estimate the frequency of infestation and not the actual number [22]. They are also a poor proxy for measuring the adult population, which is responsible for disease transmission. These indices may hence have limited use in assessing transmission risk [23].

In Singapore, the house index has been used to monitor the *Ae*. *aegypti* population in the community since the 1960s. However, the low house index of less than 1%, observed in the last few decades has rendered the house index insensitive for gauging trends in the *Ae*. *aegypti* population in Singapore [17]. As such, the house index is no longer relevant for dengue risk assessment, suggesting a need to develop new approaches to monitor the *Ae*. *aegypti* population. Expansion of surveillance tools and evaluation of populations based on adult collections were considered. As a result, a simple, hay infusion-filled cylindrical trap with a sticky lining on the inner surface was designed to lure and trap gravid female *Aedes*. The trap was named a Gravitrap to easily to define how it functions–trapping gravid mosquitoes [24]. A field trial was first conducted in a small site in 2010 to assess the effectiveness of the Gravitrap in trapping adult female *Aedes*. Subsequently, Gravitrap deployment was expanded to 34 treatment sites across the island in 2013. As part of the evolving dengue control programme in Singapore, Gravitraps have been used as a vector surveillance tool by the National Environment Agency (NEA), Singapore, to monitor the spatio-temporal variability of the adult *Ae*. *aegypti* population in Singapore [25].

In this study: we introduce a new entomological index, derived from the Gravitrap surveillance data, which provides an indication of the adult population of female *Ae*. *aegypti* in the community; the association of the index with dengue transmission risk; and the impact of large-scale deployment of such adult female traps on case counts.

## Methods

### Ethics statement

This study was granted approval by the Environmental Health Institute of the National Environment Agency, Singapore and the permission to use the dengue case data was approved by the Ministry of Health, Singapore.

### Study area and period

**Fig 1:**
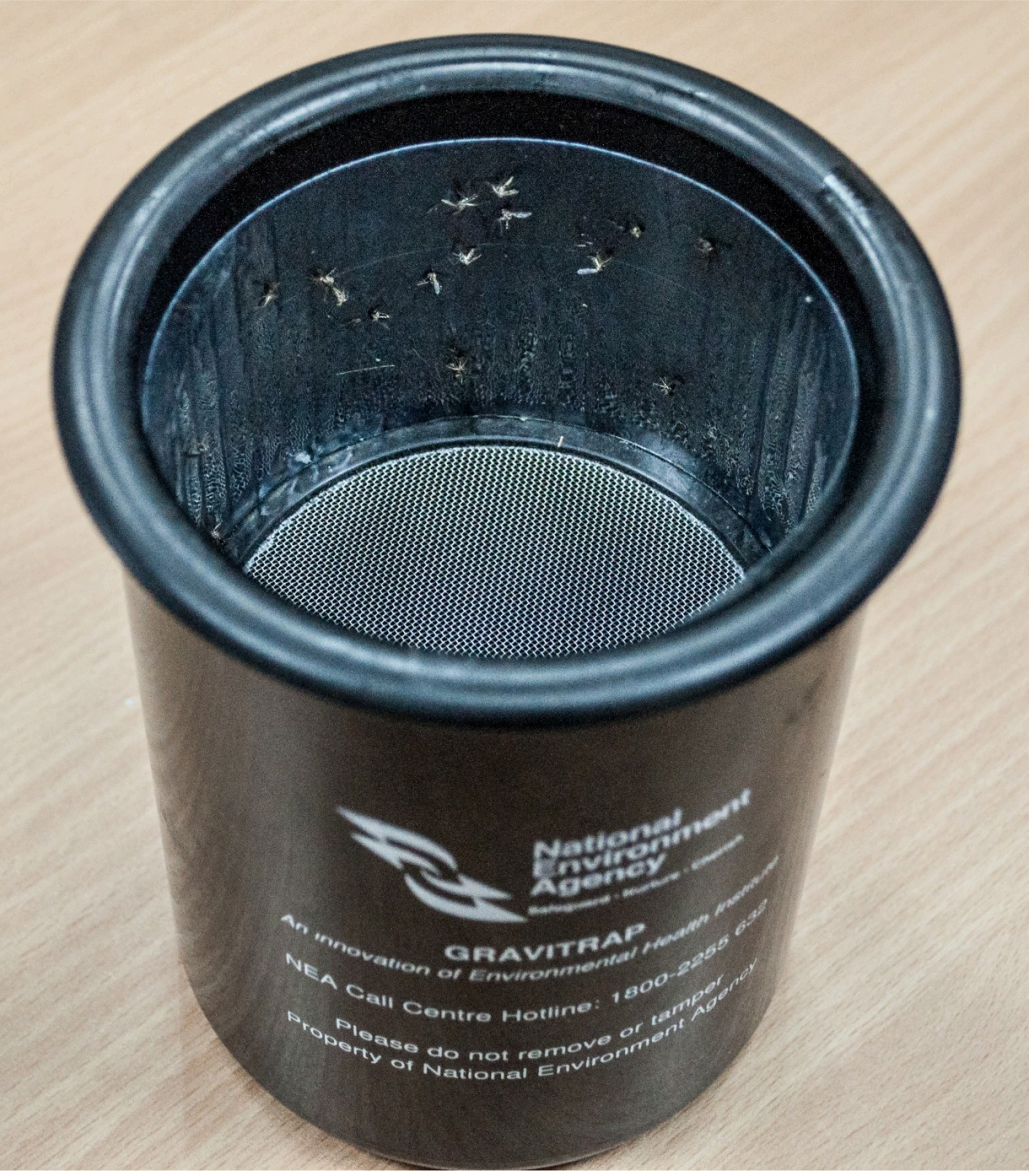
Picture of a Gravitrap with mosquitoes trapped on the sticky lining.

A total of 3,000 Gravitraps were deployed at 34 treatment sites across Singapore from September 2013 to September 2016. These treatment sites were residential areas with high rise public housing apartments. Each treatment site comprised of 5 to 30 apartment blocks that were typically 150 meters apart, 12 to 14 floors high, and with the human population ranging between 3,700 and 16,000. These sites were selected due to the high risk of dengue transmission based on a previous study that mapped the spatial risk of dengue in Singapore [26]. Gravitraps (Fig 1) were placed along the common corridors of these apartments in the ratio of 1 trap for every 20 households, i.e. two to three traps on each of three floors per apartment block: lower floor (2^nd^), mid floor (5^th^ or 6^th^ floor) and high floor (10^th^ or 11^th^ floor). This corresponded to an average of six Gravitraps per apartment block. This trap-to-household ratio and deployment was based on logistic considerations for long term monitoring, and it provided an assessment of the density as well as vertical distribution of the mosquito population. Mosquito data was collected from all 3,000 Gravitraps on a weekly basis.

### Entomological data

#### Gravitrap *aegypti* index

The Gravitrap contained a hay infusion solution, which acts as a lure for gravid female *Aedes* mosquitoes. The hay infusion solution was prepared from a stock solution consisting of 100g of dried Bermuda hay (*Cynodon dactylon*), purchased from a local equine care product supplier, for every 10L of water. The stock solution was then left at ambient temperature to ferment for 7 days, after which it was filtered and diluted by five times using water. Bacillus thuringensis israelensis (VectorBac WG, Valent BioSciences) was added accordingly to label instruction to kill immatures. During each weekly maintenance of traps, field officers removed all mosquitoes caught on the sticky lining and placed them into vials. The Gravitraps were topped up to the overflow holes with fresh working solution and all debris was removed. Gravitraps were emptied and replaced with fresh solution every eight weeks. All mosquito samples were sent to the laboratory for species identification using mosquito identification keys [27]. Information on the mosquito species, counts and locations were recorded and updated into the study database.

Gravitrap *aegypti* index (GAI), derived from the Gravitrap surveillance data, was defined as the mean number of female adult *Ae*. *aegypti* caught per functional Gravitrap. It estimates the *Ae*. *aegypti* population by normalizing the total number of female adult *Ae*. *aegypti* caught with the total number of functional Gravitrap recovered.

$$Gravitrap\;aegypti\;Index\;(GAI)=\frac{Total\;number\;of\;female\;adult\;Ae.aegypti}{Number\;of\;functional\;Gravitrap}$$

A functional Gravitrap is defined as a fully-assembled Gravitrap where the inner lining remained sticky to capture mosquitoes and hay infusion solution was present. Traps that were empty, missing, overturned, or had lining removed were considered non-functional and excluded.

#### Breeding percentage

Breeding Percentage (BP), derived from routine inspection data, is an index previously developed to estimate the proportion of *Ae*. *aegypti* positive breeding sites relative to that of total *Aedes* spp. positive breeding sites, which includes both *Ae*. *aegypti* and *Ae*. *albopictus*, the latter being ubiquitous in Singapore [28]. Larvae collected from breeding sites were sent to the laboratory for species identification using mosquito identification keys. BP is calculated from the number of *Aedes* mosquito breeding sites recorded during ground inspections carried out by NEA using the formula:

$$Breeding\;Percentage\;(BP)=\frac{No.of\;Ae.aegypti\;positive\;breeding\;sites}{No.of\;Aedes\;spp.positive\;breeding\;sites}$$

NEA carried out routine inspection surveillance across Singapore throughout the year. These inspections include those scheduled for regular preventive surveillance, and those conducted in response to dengue transmission in a location. The relationship between BP and GAI was assessed by Pearson’s correlation.

### Dengue case data

Under the Infectious Diseases Act, it is mandatory for all medical practitioners and clinical laboratories to notify all clinically diagnosed and laboratory confirmed dengue cases to the Ministry of Health [29]. The residential address and onset date of each dengue case were recorded and shared with NEA for vector control. Geo-referenced data on dengue cases were extracted from NEA’s database and anonymized prior to analysis.

### Statistical analysis

A Before-after control impact (BACI) statistical design was used to examine the impact of Gravitraps on dengue transmission. BACI designs are an effective method that is commonly used for environmental impact assessment [30,31]. The BACI approach is designed to compensate for spatial differences between the treatment and control sites, as well as temporal variance [32,33]. Two control sites were identified for each treatment site, after the selection of the treatment sites. The control sites were epidemiologically matched and randomly selected. They were of the same premise type (i.e. High-rise public housing apartments) and had the same risk of dengue transmission (i.e. High risk) as the treatment sites based on a spatial dengue risk map [26]. The absolute number of dengue cases reported is not a fair measurement of case burden in the treatment and control sites as it is often influenced by island wide dengue outbreak and thus, cannot be compared across time. Therefore, case ratio, a more robust measurement of case burden, was obtained by normalising the number of dengue cases in the treatment and control sites with the total national dengue cases. Monthly case ratios were computed for the treatment and control sites. The case ratios were analysed by fitting a linear mixed effects model using restricted maximum likelihood to estimate the effect of Gravitraps on dengue incidence. The model estimates the main effects and interaction between Site (Treatment vs. Control sites) and Period (Before vs. after Gravitrap deployment), with a random effect of Year. A statistically significant interaction between Site and Period is strong evidence for an effect of Gravitraps on dengue incidence. Data from January 2010 to December 2012 was taken as the before period (i.e. prior to Gravitrap deployment) and January 2014 to September 2016 as the after period (i.e. during Gravitrap deployment) in the BACI analysis.

All statistical analyses were performed using R software version 3.1.1 [34]. Significant differences between groups were determined by non-overlapping 95% confidence intervals. Dengue case data from January 2010 to September 2016 and Gravitrap surveillance data from January 2014 to September 2016 were used in this study.

## Results

### Temporal analysis

**Fig 2:**
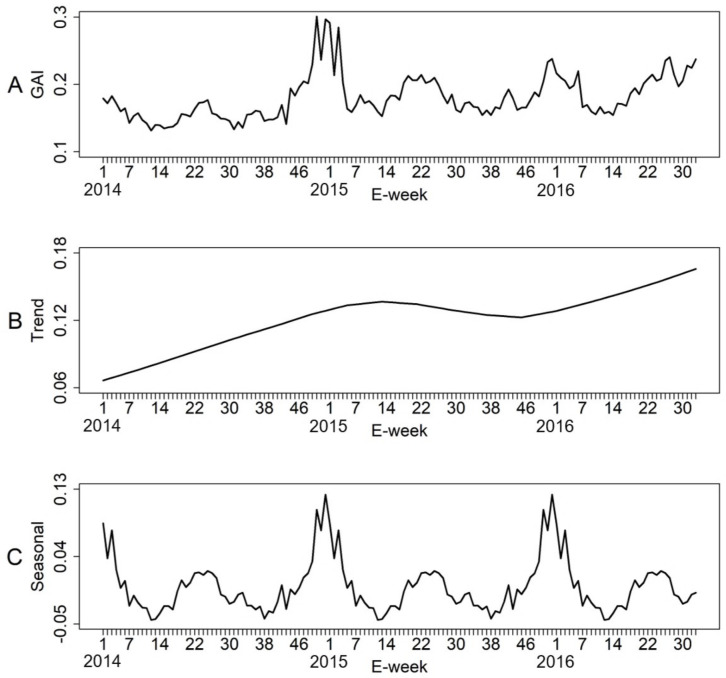
Temporal trend of the weekly Gravitrap *aegypti* Index (GAI) from 2014 to 2016 (A). Trend (B) and seasonal (C) decomposition of the GAI in Singapore.

Fig 2A shows the temporal trend of the weekly GAI of the 34 treatment sites. The GAI ranged between 0.05 and 0.30 and averaging at 0.12. The time series data was further decomposed into trend and seasonality components. As illustrated in Fig 2B, there was a gradual increase in the index since 2014. The mean GAI in 2015 (0.13) and 2016 (0.15) was 30% and 50% higher than that of 2014 (0.10) respectively. This increase indicates higher *Ae*. *aegypti* activity within the community. The seasonality component suggests a seasonal pattern in the *Ae*. *aegypti* activity (Fig 2C). The lowest activities of *Ae*. *aegypti* occurred in March (E-week 11–14) and September (E-week 37–40), coinciding with the low rainfall seasons. Peak activity, which occurred in May/June (E-week 19–26) and in December (E-week 49–52), coincided with the high rainfall seasons (S1 Fig).

### Spatial analysis

**Fig 3:**
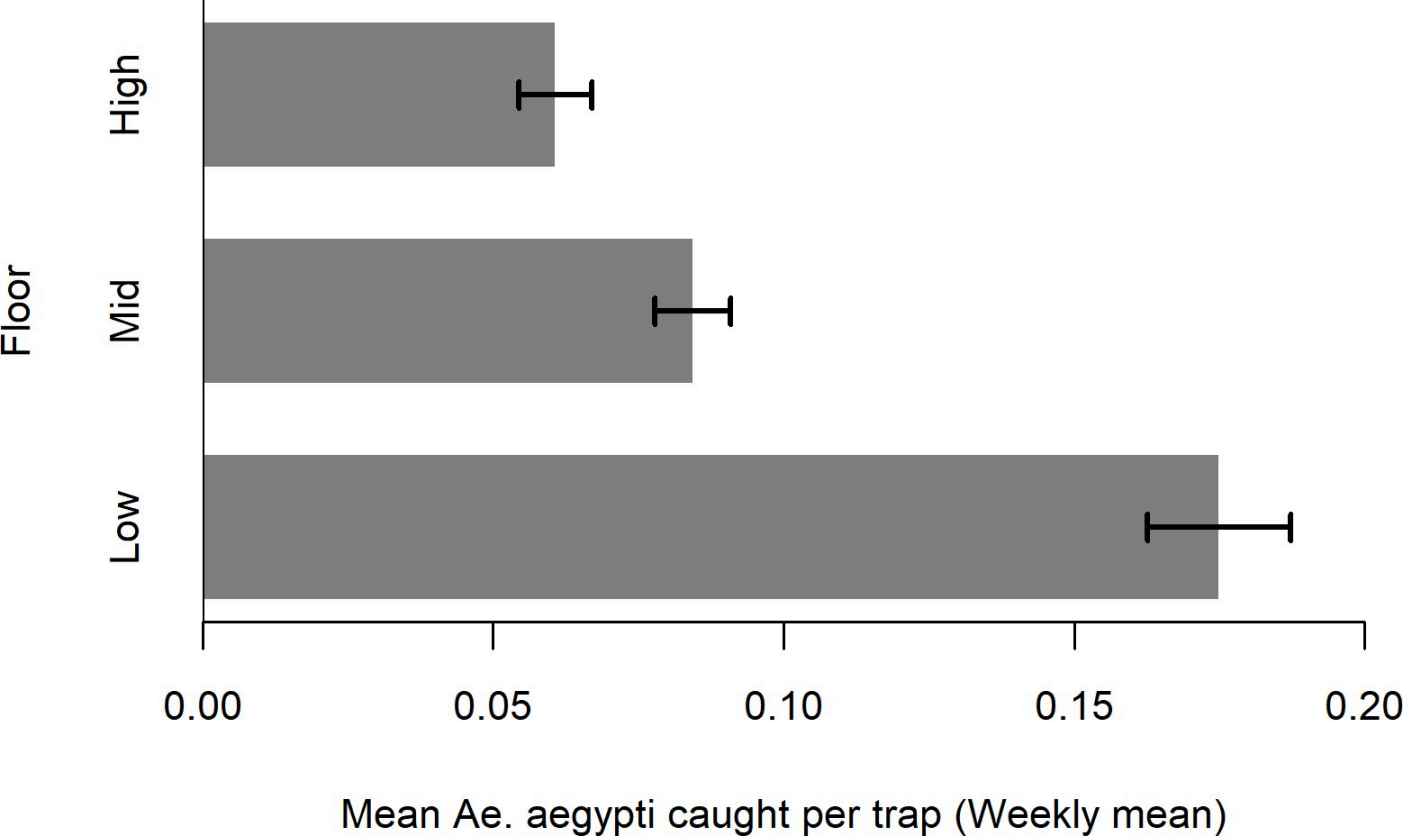
The mean *Ae*. *aegypti* caught per trap by aggregated floor (Low, Mid and High), and its 95% confidence intervals.

**Fig 4:**
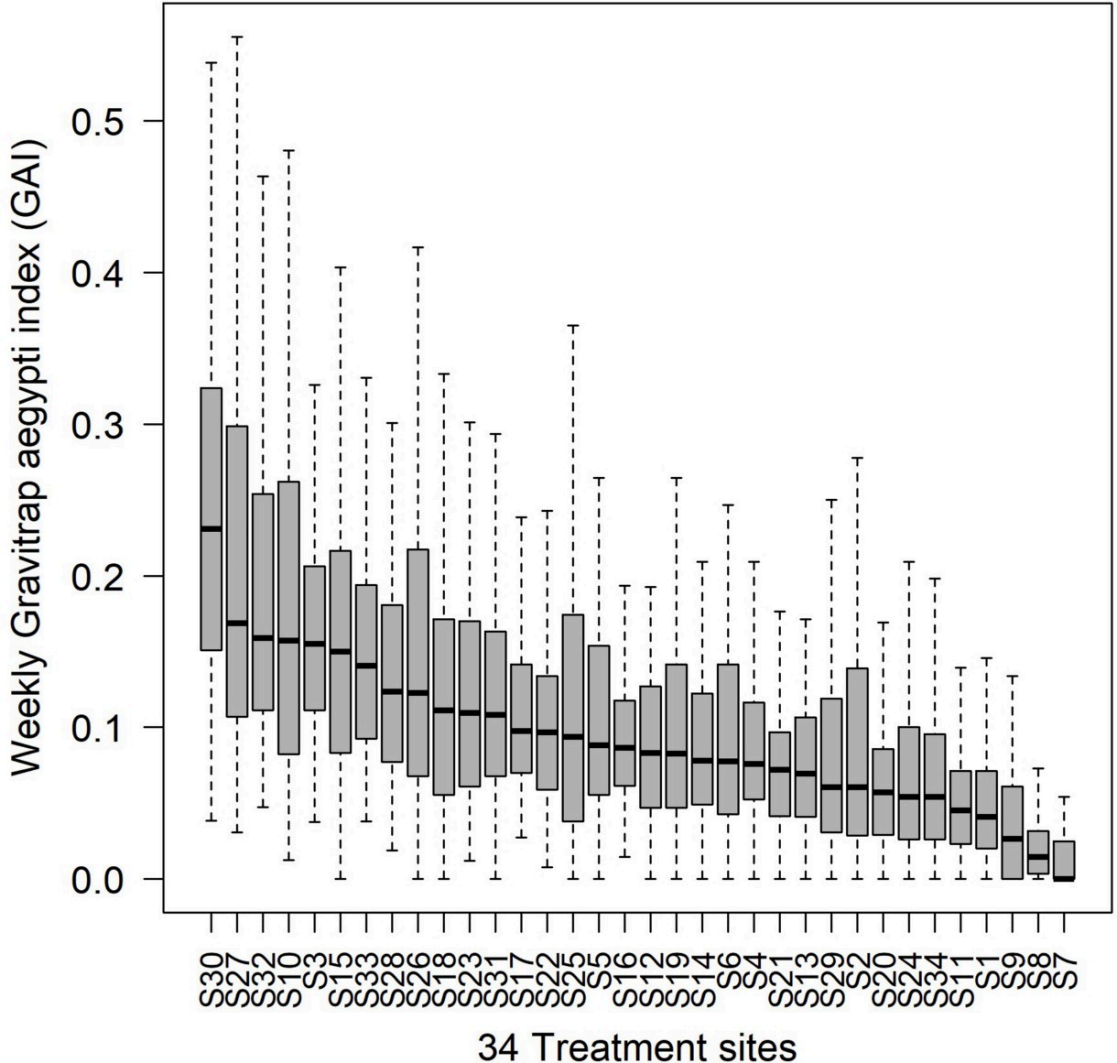
Distribution of the weekly GAI from January 2014 to September 2016 in each treatment site, arranged in descending median GAI.

Vertical distribution of the *Ae*. *aegypti* was examined, and the number of *Ae*. *aegypti* caught in the Gravitraps was found to be unevenly distributed among different floors in the apartment block, with low floors (floors 1–4) having a higher abundance of mosquitoes trapped than mid (floors 5–8) and high (floors ≥9) floors (Fig 3). The odds of detecting an *Ae*. *aegypti* positive Gravitrap at low floors were 2.05 (95% CI: 2.00,2.09) and 2.35 (95% CI: 2.27,2.43) times higher than the mid and high floors respectively. Spatial heterogeneity in *Ae*. *aegypti* abundance was observed when Gravitrap data were analysed separately for each treatment site (Fig 4). The *Ae*. *aegypti* population differs between the treatment sites, with some sites having a higher *Ae*. *aegypti* abundance than the others. Time series plot for each treatment site is shown in S2 Fig.

### Association of GAI with BP and dengue cases

**Fig 5:**
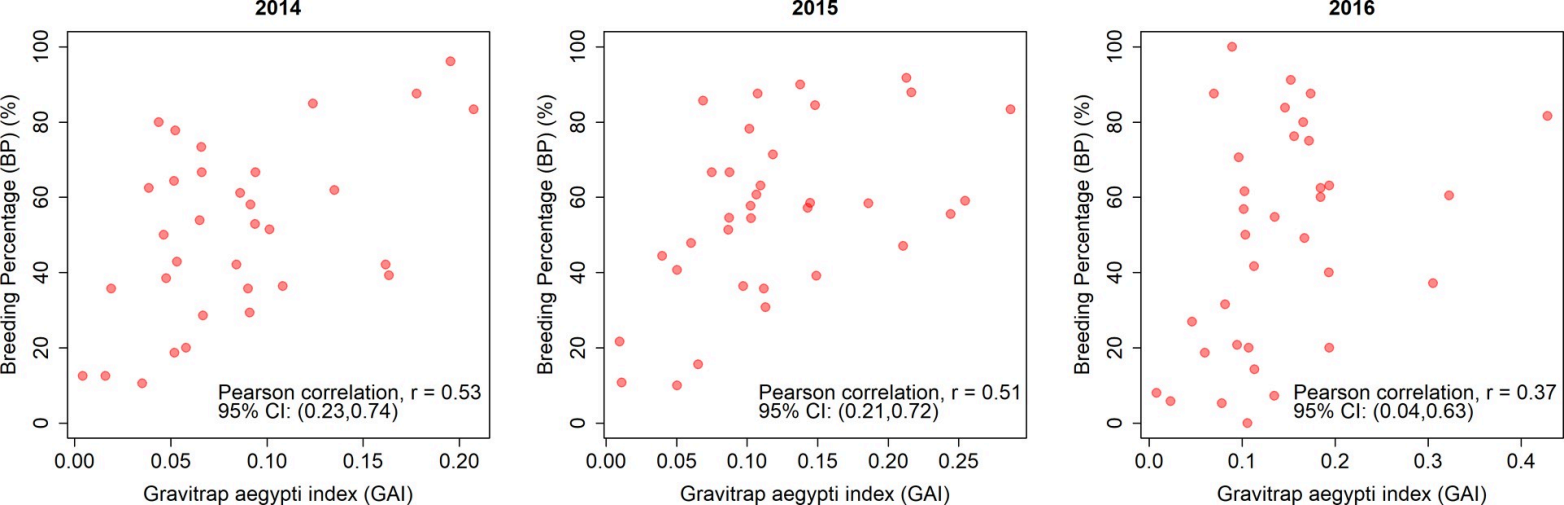
Relationship between GAI and BP assessed by Pearson’s correlation test.

**Fig 6:**
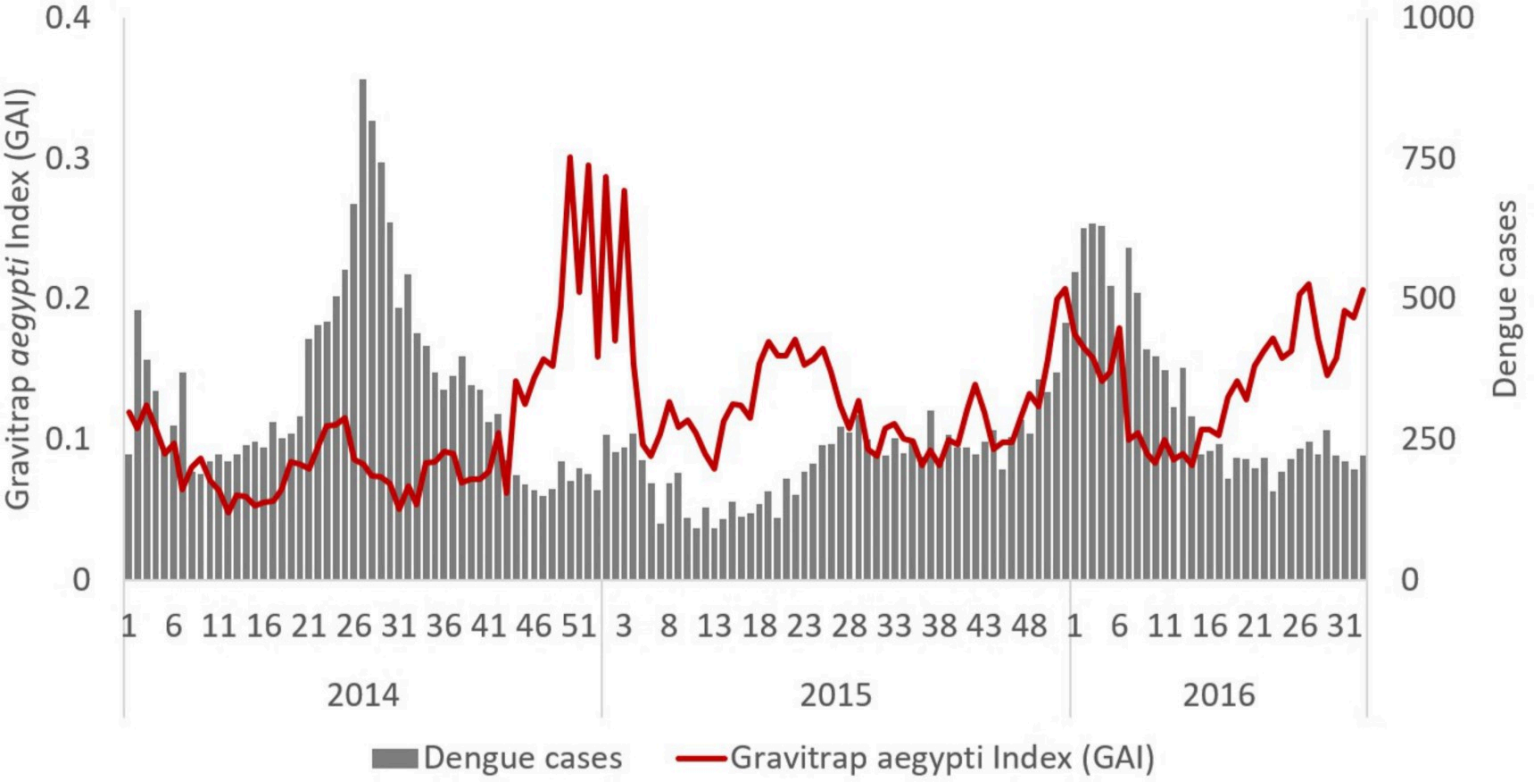
Weekly GAI and dengue cases (2014–2016).

**Fig 7:**
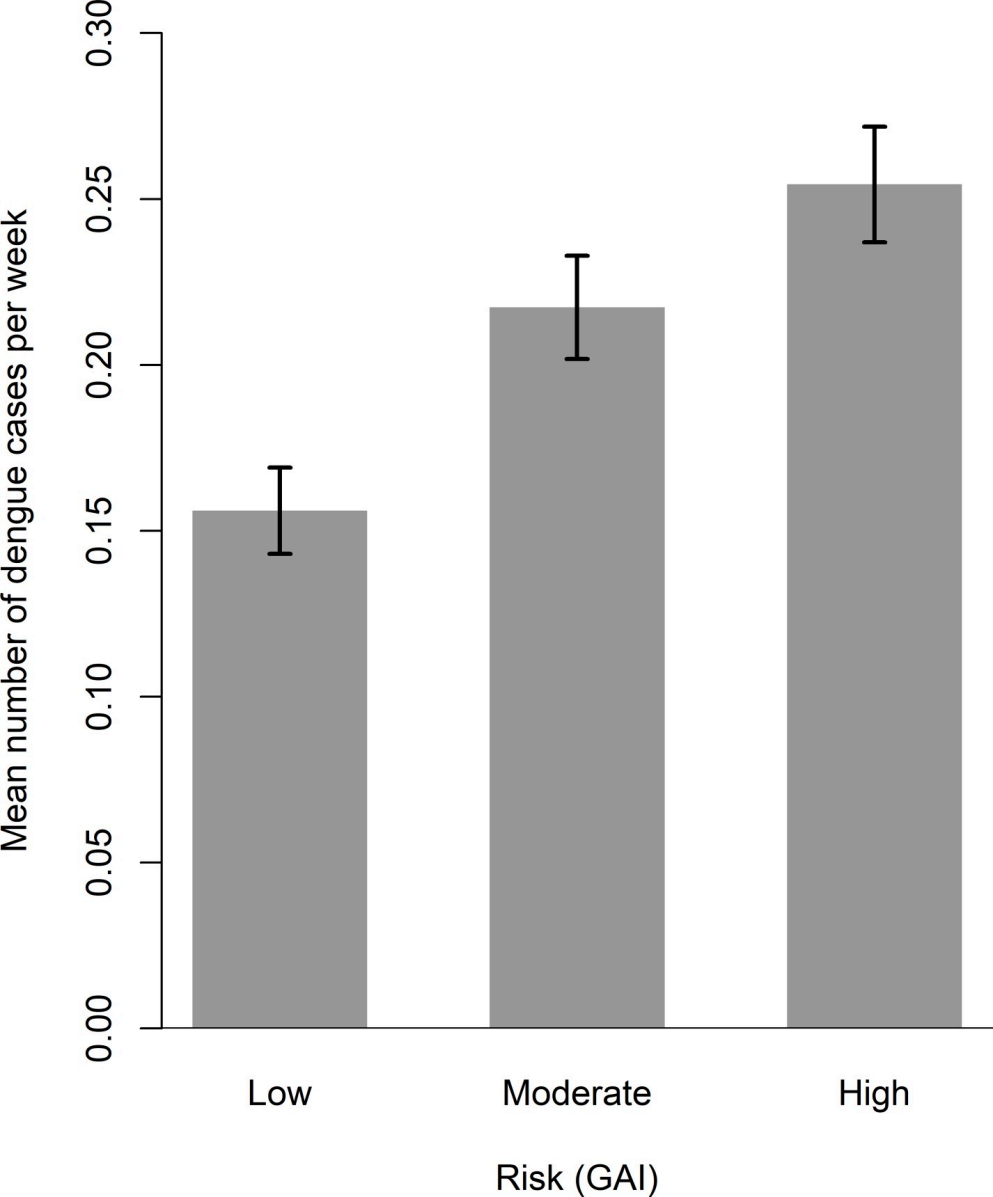
Mean number of dengue cases per week and its 95% confidence interval for each risk group (Low, Moderate and High GAI).

The GAI was consistently positively associated with BP (Fig 5). The Pearson’s correlation coefficient ranged between 0.37 and 0.53. Areas with high GAI tended to have high BP, and areas with high BP were previously shown to be associated with high risk of dengue transmission [28]. Temporal relationship between GAI and dengue cases was examined using correlation analysis. As shown in Fig 6, there was no observed temporal relationship (*ρ* = -0.14, P = 0.09) between GAI and dengue cases. However, when the GAI and dengue case data were analysed spatially at the treatment site level, it was found that treatment sites with high GAI were associated with a higher risk of dengue transmission, as indicated by the higher mean number of dengue cases per week (Fig 7). The GAI were categorized into low, moderate, and high-risk groups based on terciles, and the mean number of dengue cases per week were 0.156 (95% CI: 0.143;0.168), 0.217 (95% CI: 0.201;0.232) and 0.253 (95% CI: 0.236;0.271) respectively.

### BACI analysis

**Fig 8:**
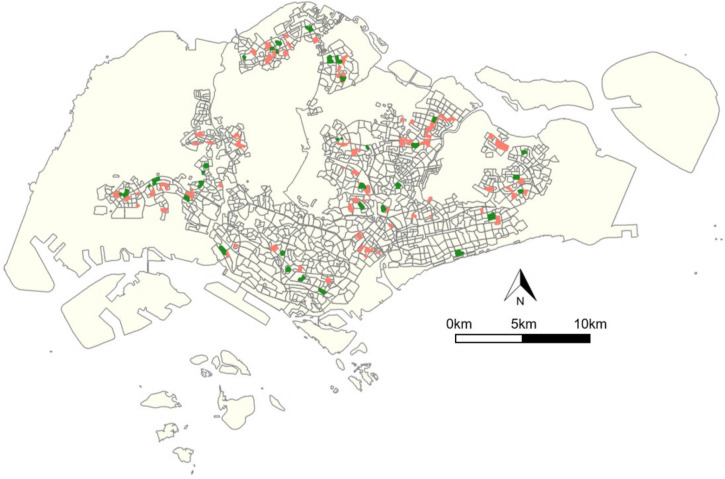
Map showing the spatial distribution of control (red) and treatment (green) sites. The grey borders indicate the residential areas in Singapore. The figure was created using R software with base layer obtained from https://www.onemap.sg/main/v2/.

**Fig 9:**
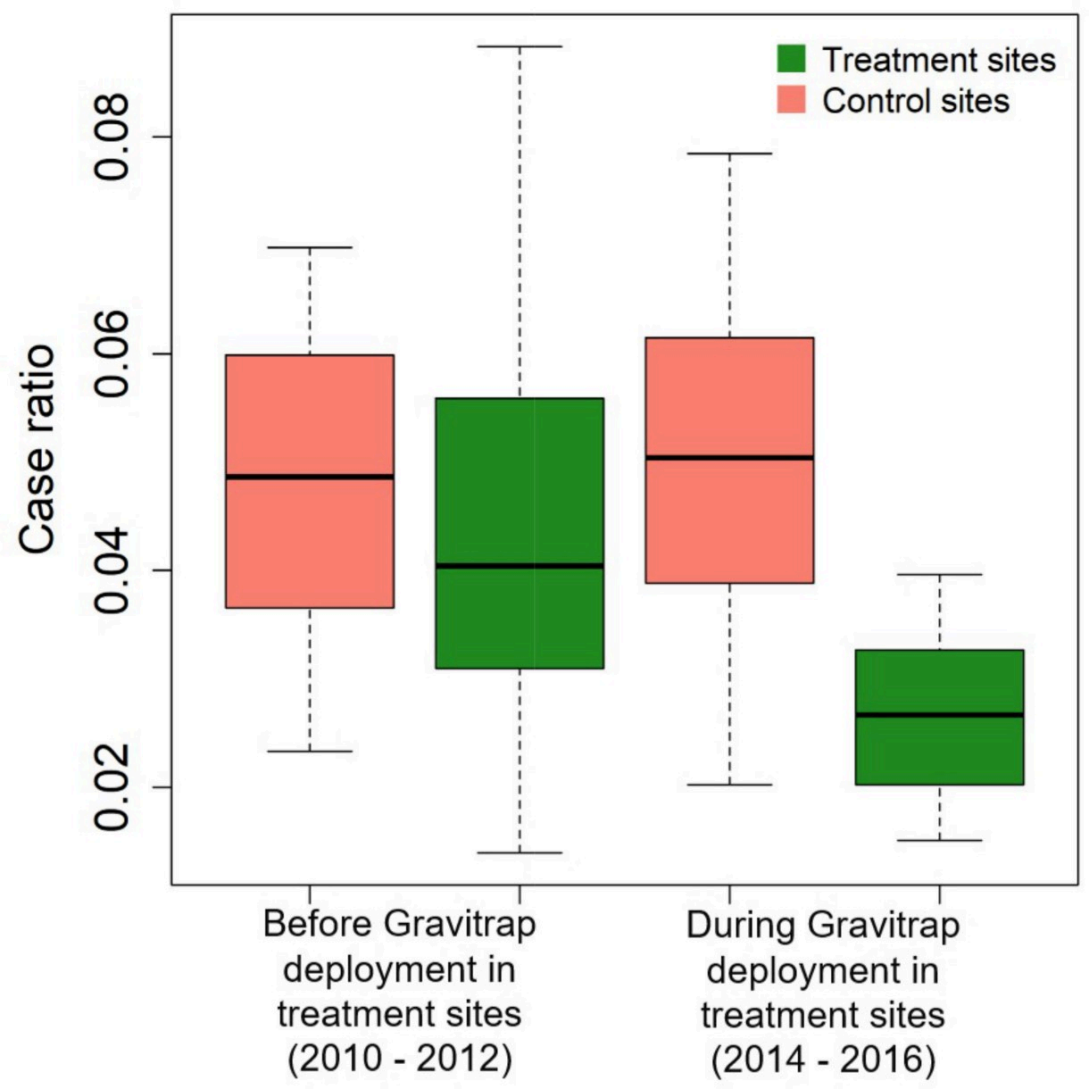
Monthly case ratios in control (red) and treatment (green) sites, before (2010–2012) and during (2014–2016) Gravitrap deployment.

Fig 8 shows the locations of the treatment and control sites. The treatment and control sites are well distributed across the country and thus nationally representative. Comparison of the before and after case ratios in sites with Gravitraps showed an impact on dengue transmission. While case ratios in control sites remained unchanged over time, the treatment sites saw a reduction in case ratio, suggesting that contribution of cases by the treatment sites to the national numbers were lower after Gravitraps were deployed (Fig 9). Results from BACI analysis were summarized in Table 1. The main effect terms, Site and Period, were both non-significant. Case ratios in treatment and control sites were of no difference prior to Gravitrap deployment and there were no changes in control sites’ case ratios over time. The interaction term, on the other hand, suggested evidence of treatment effect of Gravitraps. There was a 0.017 reduction in treatment sites’ case ratio after Gravitraps deployment. This corresponds to a 36% reduction in case burden in the treatment sites when comparing to the control sites.

**Table 1:** Output of linear mixed model with Site and Period as categorical variable and product of Site and Period (interaction term) entered to the model. Outcome is the monthly case ratios.

				95% CI	
	Estimate	Std. Error	P-value	Lower	Upper
**Intercept**	0.046	0.003	<0.001	0.040	0.052
**Site**	-0.005	0.003	0.138	-0.011	0.001
**Period**	0.006	0.005	0.265	-0.004	0.016
**Site:Period**	-0.017	0.005	<0.001	-0.027	-0.007

## Discussion

Dengue is transmitted by the adult female *Aedes* mosquito. The collection of the adult female *Aedes* mosquito is thus important to understand disease transmission dynamics. Information on its distribution and density are also essential for assessing transmission risk as well as for devising an effective control strategy [35]. Surveillance of the adult *Aedes* mosquito would therefore be more appropriate for risk assessment than larval indices, whose association with dengue transmission have yet to be proven satisfactory [36]. In recent years, several adult traps (e.g. BG-Sentinel, MosquiTrap, etc.) have been developed to address the increasing need for an efficient adult collection, for both surveillance and control. Even though their efficacy in trapping adult *Aedes* mosquitoes has been demonstrated in many studies, their utility is limited due to various setbacks such as cost, size, and dependence on electric power [37–40]. Most importantly, what is still lacking is the development of an accurate method of estimating the adult *Aedes* mosquito density based on the trapping data. Despite the limitations, these adult traps have been useful for entomological studies but are inadequate for epidemiological studies.

This paper described a new approach to dengue vector surveillance, introduced a new entomological index that estimates the adult population of *Ae*. *aegypti* in the environment and presented its association with dengue transmission risk. The GAI, provides a measure of the *Ae*. *aegypti* density by normalizing the total number of female adult *Ae*. *aegypti* caught with the total number of Gravitraps. Unlike traditional indices such as House Index, Container Index and Breteau Index, which are reported on a national level or a neighbourhood level, the main strength of the GAI is its higher resolution in time and space. This is largely due to the possibility of deploying large number of low-cost traps for longitudinal monitoring survey. Depending on operational needs, the index can be computed at different spatial scales. Data can be aggregated to estimate the *Ae*. *aegypti* population in the country or analysed separately to estimate the population within a predefined area.

Spatio-temporal patterns of the *Ae*. *aegypti* population can be examined to assist the planning of vector control. In our study, time series data of the GAI revealed an increased *Ae*. *aegypti* activities over the years. The *Ae*. *aegypti* population exhibits a seasonal pattern, characterized by two troughs and peaks. The first trough occurs in March but rises to a peak in May. The *Ae*. *aegypti* population decreases subsequently to form a second trough in September and rises again to a peak in December. A recent study on the seasonal fluctuations of *Ae*. *aegypti* larval and pupae in Singapore also revealed a bimodal seasonal pattern which peaks in April and November [41]. The consistent one-month lag in the peaks may be attributed to the development and longevity of the *Ae*. *aegypti* [42]. Spatially, *Ae*. *aegypti* is most abundant on the lower floors of high-rise housing apartments, consistent with those of previous studies that had found that *Ae*. *aegypti* prefer to oviposit near ground level [43,44]. This indicates that vector control efforts should be prioritized at lower floors given limited resources. Spatial heterogeneity in *Ae*. *aegypti* populations were observed among treatment sites, a possible contributing factor to the differential transmission risk between areas.

Unlike house inspection, Gravitraps which lure mosquitoes are not biased by the inaccessibility of premises or cryptic breeding sites. Since the implementation of Gravitraps in 2013, the GAI has been used as the national vector index for monitoring the spatio-temporal variability of the adult *Ae*. *aegypti* population in Singapore, replacing the previously used house index, which is no longer sensitive for dengue risk assessment. Our study showed that areas with high GAI were associated with higher dengue case count. Therefore, the GAI can be used as a risk factor and be incorporated into dengue forecast models as well as the spatial dengue risk map, that had been developed to predict dengue incidence and to guide the prioritization of resources respectively [26,45,46]. Besides being an efficient surveillance tool, Gravitrap appears to have an impact on dengue transmission, hypothetically due to the removal of infected mosquitoes. This is consistent with the recent study by Barrera et al., which shows that adult mosquito traps can protect humans from disease acquired from infected *Ae*. *aegypti* mosquitoes [47].

Under the islandwide Gravitrap surveillance programme, NEA has progressively deployed Gravitraps across all apartment blocks in Singapore since 2017, with more than 50,000 Gravitraps deployed across 9,000 apartment blocks currently. The GAI data has been used to alert stakeholders and community to proactively reduce mosquito breeding [48–50]. Areas with high GAI received more house inspections. Information on areas with high GAI is also made publicly available through NEA website and myENV app (an application by NEA), to nudge the community to conduct pre-emptive source reduction even before any report of dengue transmission [51,52].

The re-emergence of dengue fever in many countries, despite low immature indices, has warranted the need for a more effective index for dengue vector surveillance and control. The GAI, which is more relevant for dengue risk assessment, is recommended as an indicator for decision making in vector control efforts.

## Supporting information

S1 Fig Monthly rainfall (mm) in Singapore (2014–2016). (TIF)

S2 Fig Temporal trend of the weekly GAI of each treatment site (2014–2016). (TIF)
