# Supplementary figures and images for: Gravitrap deployment for adult Aedes aegypti surveillance and its impact on dengue cases
Source: PLoS Negl Trop Dis. 2020 Aug 7;14(8):e0008528. doi: 10.1371/journal.pntd.0008528 (PMC7439811; doi:10.1371/journal.pntd.0008528)

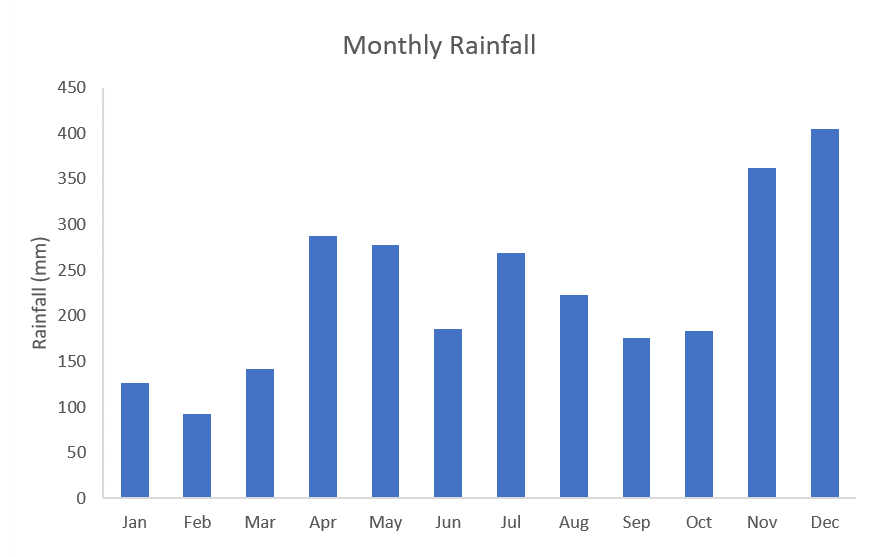

Supplement: S1 Fig — (TIF) [file pntd.0008528.s001.tif]

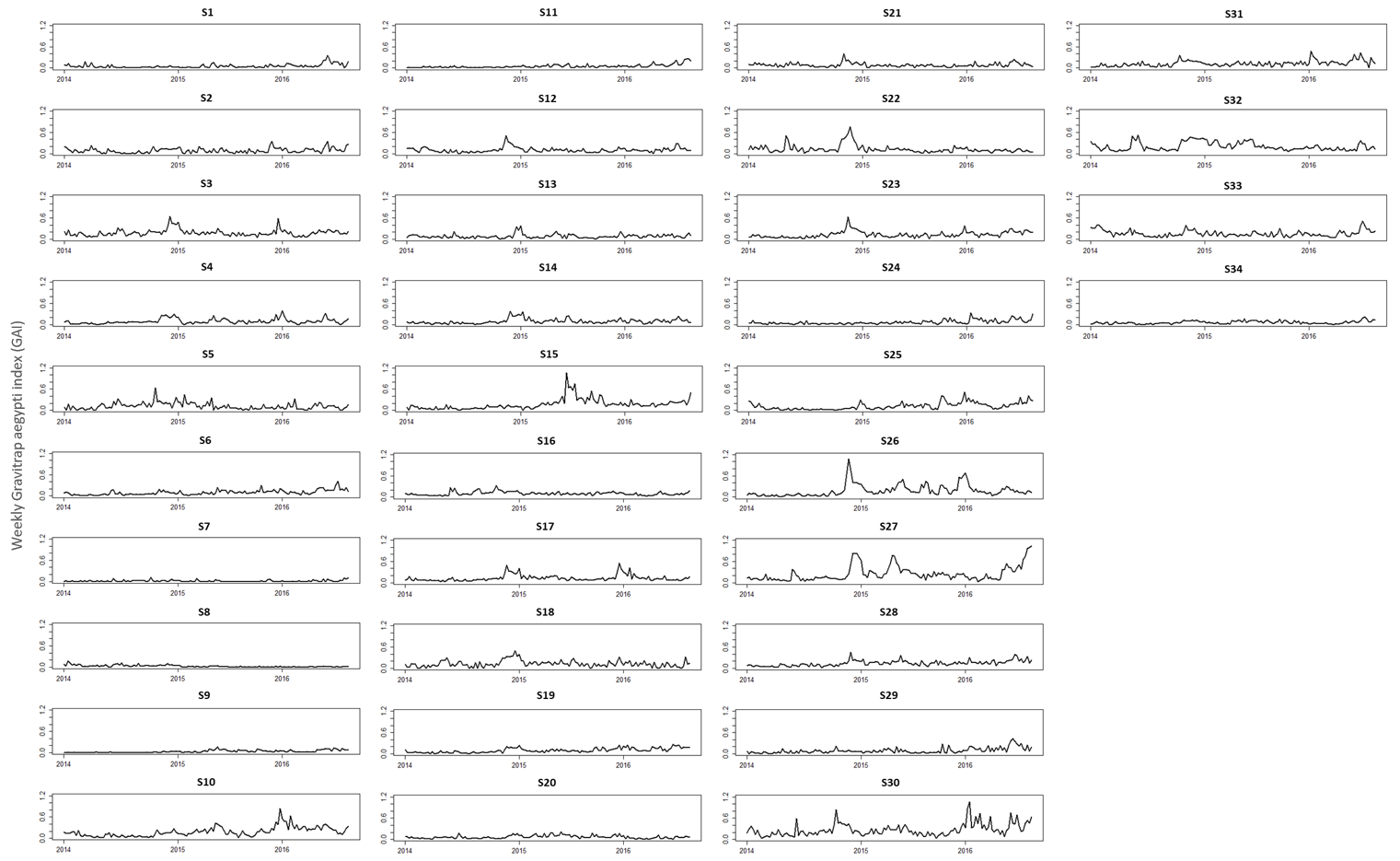

Supplement: S2 Fig — (TIF) [file pntd.0008528.s002.tif]
